# A Beckwith-Wiedemann syndrome case with de novo 24 Mb duplication of chromosome 11p15.5p14.3

**DOI:** 10.1186/s13039-021-00532-7

**Published:** 2021-03-03

**Authors:** Huling Jiang, Zepeng Ping, Jianguo Wang, Xiaodan Liu, Yuxia Jin, Suping Li, Chiyan Zhou, Pinghua Huang, Yi Jin, Ling Ai, Jie Chen

**Affiliations:** 1 Department of Prenatal Diagnosis Center, Maternity and Child Health Care Affiliated Hospital, Jiaxing University, Jiaxing, 314000 China; 2 Department of Pediatric Surgery, Xinhua Hospital Affiliated To Shanghai Jiao Tong University School Of Medicine, Shanghai, 200092 China

**Keywords:** Beckwith-wiedemann syndrome (BWS), Chromosome 11p15.5, Paternal duplication, Single Nucleotide polymorphism (SNP) array analysis, Methylation-specific multiplex ligation-dependent probe amplification (MS-MLPA)

## Abstract

**Background:**

Molecular genetic testing for the 11p15-associated imprinting disorder Beckwith-Wiedemann syndrome (BWS) is challenging because of the molecular heterogeneity and complexity of the affected imprinted regions. An integrated molecular approach to analyze the epigenetic-genetic alterations is required for accurate diagnosis of BWS.

*Case presentation*: We reported a Chinese case with BWS detected by SNP array analysis and methylation-specific multiplex ligation-dependent probe amplification (MS-MLPA). The genetic analysis showed a de novo duplication of 24 Mb at 11p15.5p14.3 is much longer than ever reported. MS-MLPA showed copy number changes with a peak height ratio value of 1.5 (three copies) at 11p15. The duplication of paternal origin with increase of methylation index of 0.68 at *H19* and decreased methylation index of 0.37 at *KCNQ1OT1*.

**Conclusion:**

Combined chromosome microarray analysis and methylation profiling provided reliable diagnosis for this paternally derived duplication of BWS. The phenotype associated with 11p15 duplications depends on the size, genetic content, parental inheritance and imprinting status. Identification of these rare duplications is crucial for genetic counselling.

## Introduction

Beckwith-Wiedemann syndrome (BWS) (OMIM#130,650) is an imprinting disorder characterized by variable presence of macroglossia and over-growth, abdominal wall defects, hypoglycemia and embryonal tumor predisposition [1, 2]. Although BWS might present prenatally or in adult life, it is most commonly diagnosed in the neonatal period or in early childhood, with an estimated prevalence of one affected child per 10,340 live births [1]. BWS is caused mainly by genetic or epigenetic defects within the evolutionary conserved imprinted gene cluster located at chromosome 11p15.5 region [3] which consists of two imprinted domains, *H19/IGF2*:IG-DMR(IC1) and *KCNQ1OT1*:TSS-DMR(IC2) [4, 5]. Among several causative alterations identified so far, gain of methylation (GOM) at *H19/IGF2*:IG-DMR (5–10%), loss of methylation (LOM) at *KCNQ1OT1*:TSS-DMR (50%) which leads to biallelic expression of *KCNQ1OT1* and biallelic silencing of *CDKN1C*, and 11p15 paternal uniparental disomy (UPD) (20%), loss-of-function pathogenic variants of *CDKN1C* (5%), and 11p15.5 copy-number variants (CNVs, 1–4%) are isolated epi-mutations [6]. Although most cases are sporadic, about 5–10% of BWS patients have inheritance characteristics [7]. We investigated a patient with clinical features of BWS. Genetic analyses revealed that the patient harbored pUPD and copy number variation. This is a rare reported case of a patient with 24 Mb duplication, which is much longer than ever reported.

## Materials and methods

### Patient report

The female infant was the first-born baby to non-consanguineous, healthy, Chinese parents after 36 weeks and 6 days of gestation in Jiaxing maternal and Child Health Hospital. Her birth weight was 3790 g, and she exhibit edcolumnar head, collapse of nasal bridge, wide distance of eyes, right hand through the palm, mild tricuspid regurgitation (Opposite velocity: 2.88 M/S, PG:33.2 mmHg), patent ductus arteriosus, the right ventricle is markedly enlarged. Bilateral ventricle dilation (Left: body: 9.3 mm, Relief angle: 19.7 mm. Right: Body: 6.6 mm, Relief angle: 17.5 mm). Abdominal hydrocele (Left lower abdomen: 15 × 8 mm anechoic area. Right abdomen: 47 × 13 mm anechoic area)lower xiphoid process abdominal bulge, rectus abdominis separation, renal hypertrophy (Left kidney: 54 × 27 mm, Right kidney: 56 × 29 mm), Long QT syndrome, thickened pulmonary texture, enlarged heart shadow, pulmonary hypertension, expanded perineal anal junction, mixed echo of right lobe of liver. This research was approved by the Ethics Committee of the Jiaxing Maternity and Child Health Care Hospital.

### Genetic analyses

#### DNA isolation

Sample was collected and genomic DNA was extracted from the peripheral blood of the patient. Isolation and purification of the genomic DNA were performed using a Qiagen DNeasy Tissue Kit according to the manufacturer’s instructions (Qiagen, Hilden, Germany).

#### SNP array analysis

Single nucleotide polymorphism (SNP) array analysis was performed with CytoScan 750 K/HD array (Carlsbad, CA, USA) according to the manufacturer’s protocol. The microarray was used to investigate the CNVs and absence of heterozygosity (AOH) events.

The locations of the CNVs and the UPD events were determined based on a human genome assembly from February 2009 (GRCH37/h19). OMIM genes and Ref-Seq genes were used to evaluate the CNVs identified in this study. The criteria used for interpreting whether a CNV was pathogenicor benign were according to the guidelines recommended by the American College of Medical Genetics (ACMG) [8].

#### MS-MLPA

Methylation-specific multiplex ligation-dependent probe amplification (MS-MLPA) was carried out as a parallel test to confirm the variations observed by SNP array. The MS-MLPA probe (ME030 for BWS/SRS, MRC-Holland, Amsterdam, The Netherlands) was used to detect copy number changes and to analyze CpG island methylation statuses of the 11p15 region. The MS-MLPA procedure was performed according to the manufacturer’s instructions. The products were detected via capillary electrophoresis using an ABI-3100 genetic analyzer (Applied Biosystems) and were analyzed using Gene Marker software (SoftGenetics, Pa., USA).

## Results

### Identifying Pathogenic CNVs via SNP array analysis

**Fig. 1:**
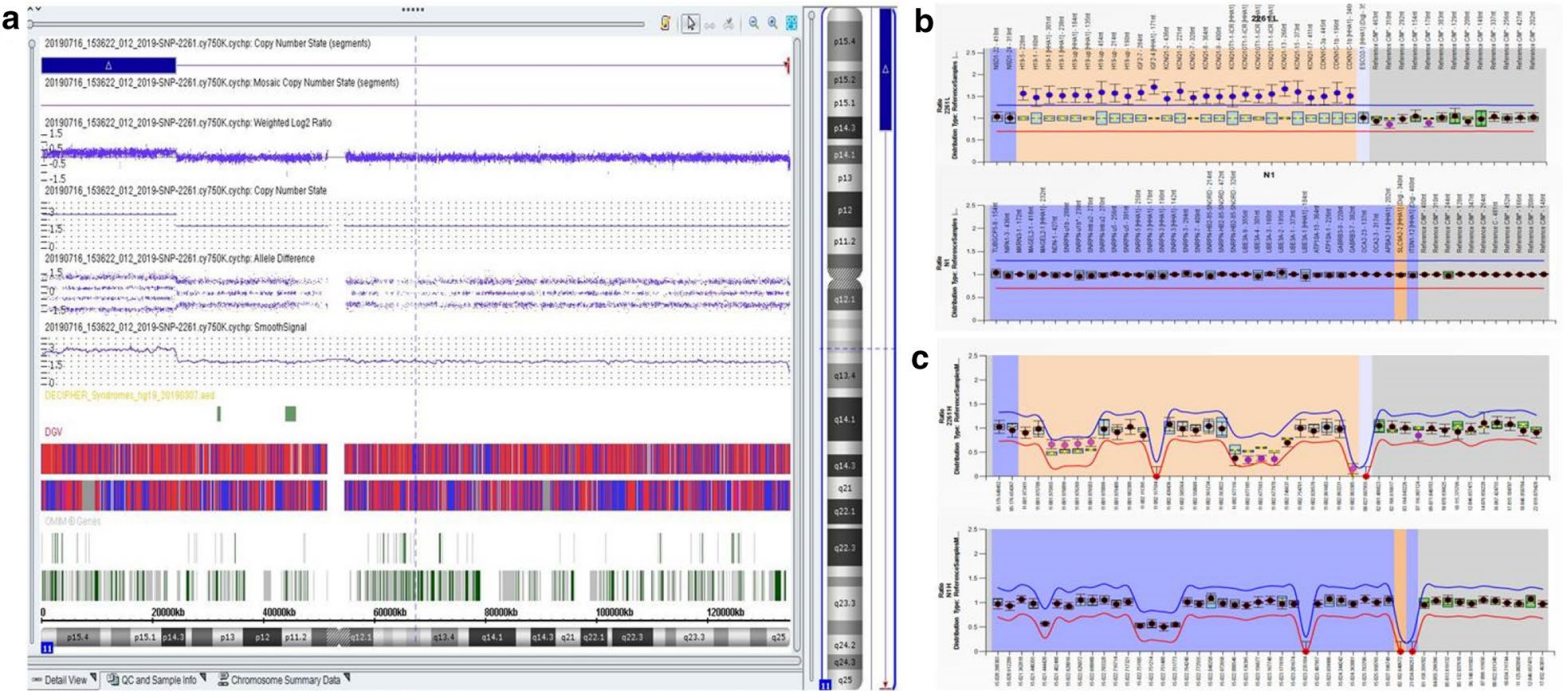
**a** The SNP array revealed a 24,170 kb duplication at (arr[hg19]11p15.5p14.3 (230,680_24,400,276)×3)). **b** MS-MLPA result of copy number change. **c** MS-MLPA result of methylation index

The SNP array result showed a 24,170 kb duplication at 11p15.5p14.3 including genes from *DEAF1* to *FANCF*(arr[hg19]11p15.5p14.3 (230,680_24,400,276)×3) (Fig. 1a).

### MS-MLPA Analysis

MS-MLPA analysis was used to validate the results observed with CMA. MS-MLPA for 11p15.5 showed copy number changes with a peak height ratio value of 1.5 (three copies) at 11p15 (Fig. 1b) in comparison with a ratio value of 1 (two copies) from a normal control. MS-MLPA indicated methylation index of 0.68 at IC1 and methylation index of 0.37 at IC2 (Fig. 1c). We indentified this patient with duplication of the BWS critical region.

## Discussion

In this study, we report a Chinese BWS case with de novo paternally derived duplication and SNP-array test revealed a 24 Mb duplication at 11p15.5p14.3, involving 210 OMIM genes. It’s a pity that we did not perform a peripheral blood chromosome examination. A number of paternal reciprocal translocations associated with 11p15.5 duplications in the affected children have been reported [9–13]. It has been reported that patients with BWS due to a paternally inherited 11p15.5 duplication exhibit macroglossia, distinct craniofacial features, including prominent occiput and forehead, a round face with full cheeks, broad and flat nasal bridge, micrognathia, hypertelorism as well as deep set eyes with epicanthus.

Although our patient’s clinical phenotype fits well to this description, and the results from SNP array and MS-MLPA analysis fulfilled the diagnostic criteria for BWS, the 24 Mb duplication at 11p15.5p14.3 is much longer than ever reported [14, 15]. Qin Wang reported two Chinese cases with BWS, One case was a de novo paternal origin duplication spanning 896 Kb at 11p15.5. Case 2 was referred at 2-month old and the genetic analysis showed a de novo 228.8 Kb deletion at 11p15.5 telomeric end and a de novo duplication of 2.5 Mb at 11p15.4p15.5. Both duplications are paternal origin with gain of methylation at the imprinting center 1 [13]. In our case, the de novo duplication of 24 Mb is much longer and involving more genes.

We consider that most of the symptoms in our patient is caused or modulated by the duplication of these genes, including the OMIM genes *H19, IGF2, TH, KCNQ1, STIMI* and so on, involving 210 OMIM genes. *H19* (103,280) plays a key role in the development of Beckwith-Wiedemann syndrome, Silver-Russell syndrome and it had been hypothesized that loss of *H19* expression may be involved in Wilms tumorigenesis [16]. *H19* is a developpmentally regulated gene with putative tumor suppressor activity. *IGF2* (147,470) is a protein hormone involved in the regulation of cell proliferation, growth, migration, differentiation, and survival. It has been found that aberrant processing of pro-IGF2 by PCSK4 may be a cause of intrauterine growth restriction, a leading cause of perinatal mortality [17]. The expression of the *IGF2* and *H19* genes is imprinted. Although these neighboring genes share an enhancer, *H19* is expressed only from the maternal allele, and *IGF2* only from the paternally inherited allele. The region of paternal-specific methylation upstream of *H19* appears to be the site of an epigenetic mark that is required for the imprinting of these genes. The *KCNQ1OT1* (604,115) gene was expressed preferentially from the paternal allele [18], while *KCNQ1* transcription is silent. In most patients with BWS, *KCNQ1OT1* is abnormally expressed from both the paternal and maternal alleles. 21 of 36 (58%) BWS patients showed loss of maternal allele-specific methylation of a CpG island upstream of *KCNQ1OT1*. The authors determined that LOI of *KCNQ1OT1* is the most common genetic alteration in BWS [19].

Chang reported a patient with a loss of heterozygosity in the region of chromosome 11p14.3 to 11p15.5. This region is similar to the case in this article. The results suggest that paternal uniparental isodisomy of chromosomal 11p15.5 is associated with the beta-thalassemia major in the patient [20]. In this case, the hemoglobin (Hb) of 117 g/L, RBCs of 3.17 × 10 × /L, may also be associated with the beta-thalassemia. Iourov demonstrated that shorter long contiguous stretches of homozygosity (LCSH) at chromosomes 7q21.3, 7q31.2, 11p15.5, and 15p11.2 occur with a frequency of about 5% in the children with intellectual disability, autism, congenital malformations and/or epilepsy. Consequently, this type of epigenetic mutations appears to be the most common one among children with neurodevelopmental diseases [21].

In this case, because the increase of paternal gene’s copy number, the *H19* methylation index of 0.68 at IC1 is increased in comparison with normal control methylation index of 0.54 at IC1. And the *KCNQ1OT1* methylation index of 0.37 at IC2 is decreased in comparison with normal control methylation index of 0.57 at IC2. A recent study in a serial of over 400 BWS cases also indicated that copy number changes in the 11p15.5 region contributed significantly to the etiology of the BWS [6]. Macchiaiolo M suggested that vascular tumors can also be associated with BWS[22]. In this case, the enlarged heart shadow, pulmonary hypertension, expanded perineal anal junction and mixed echo of right lobe of liver may also be with BWS. Papulino suggested there is an increased incidence of childhood tumor predisposition in BWS patients [9]. Cöktü [23] identified a group of 321 individuals with a molecularly confirmed diagnosis of BWS and analysed the cancer incidence. They confirmed an increased cancer risk in children with BWS. And suggested that the highest cancer risk is associated with UPDpat. So for this patient, the cancer risk may also increase, and physical examination should be performed rotinely for potential intellectual disability and the possible clinical effect involving the deleted genes. The phenotype associated with 11p15 duplications depends on the size, genetic content, parental inheritance and imprinting status. Identification of these rare duplications is crucial for genetic counselling. Furthermore, SNP arrays can be helpful in clarifying the molecular diagnosis in patient with BWS, especially to discriminate between pUPD and duplications [24].
